# Lachesis—Its Origin and Pathogenetic Effects

**Published:** 1896-01

**Authors:** W. E. Leonard

**Affiliations:** Minneapolis


### LACHESIS—ITS ORIGIN AND PATHOGENETIC EFFECTS.

Prof. W. E. Leonard, M. D., Minneapolis.

The introduction of snake poisons, of which this drug is the type, as medicines, is entirely due to Dr. Constantine Hering, who experimented with this and other snake venoms while an agent of the German Government in South America, and first published his "Effects of Snake Poisons" in 1837, in the German language. If Dr. Hering, even admitting many errors of judgment from which no man is exempt, had done nothing more than this for the science of therapeutics, his name would be immortal in medical annals; and in this, his crown of glory, Lachesis, would be by far the brightest jewel.

Our name for this remedy is taken from the specific scientific name, viz.: Trigonocephalus Lachesis, as given the snake by Linnæus, from its common title, lance-headed viper, and Lachesis, the name of one of the Greek Fates, since the bite of the reptile was sure and swift death.

The snake is an immense one, seven feet or more long, with poison fangs of nearly an inch in length. His reddish-brown skin, marked along the back with large blackish-brown rhomboidal spots, makes Dr. Hering's specimen, now in alcohol in the American Academy of The Natural Sciences in Philadelphia, a very noticeable addition to this remarkable collection.

The poison resembles saliva, being limpid, inodorous, tasteless, and in color greenish. At the extremity of the fangs it easily forms into drops and falls without threading; exposed to the air it soon concentrates into a dry, yellow mass, which is intensely poisonous for an indefinite period.

The story of the heroism exhibited in obtaining the original and only supply of this venom was intensely thrilling, as heard by the writer seventeen years ago from Dr. Hering himself, in Saturday evening talks to a small coterie of students. When a young man of thirty-five, he and his wife were directing botanical and zoölogical collectors from a temporary dwelling in the edge of the tropical forests of the upper Amazon. The natives, who were his sole assistants, had told him much of this deadly serpent, and he offered liberal rewards for the capture of a living specimen. Finally a bamboo box was brought in hastily and placed in his rooms. Immediately to his amazement, not only those capturing the serpent, but his entire native household, fled precipitately from the place. They saw no hope for their master or his wife if he proposed to deal in any way with a living Churukuku—the native name for the reptile. He was left to obtain the venom from this creature with his wife's aid alone and at the imminent risk of his life. This was done by stunning the serpent by a heavy blow as the box was opened, then holding his head in a forked stick and obtaining the poison by pressing it out from the venom bag upon sugar of milk, all of which, with the lower preparations, have been exhausted, up to the sixth.

Thus handling and triturating the virus, with the natural fear and excitement of the adventure, threw the doctor into a fever that night with tossing, delirium, and mania. His faithful wife anxiously watched over him, alone in the forests, miles from a human being, and not daring to think of the probable issue of a struggle with such a mighty poison and with no knowledge of an antidote. Toward morning he slept, and finally awoke, his mental horizon cleared from the passing storm. His first question when a little water had moistened his throat was, "What did I do and say?" One can easily imagine that the details of such a night would not be readily forgotten by the young wife, even if she was not cool enough to take notes at the time.

Before their native help, one by one, expecting to find their corpses, crept sheepishly back to the camp, this enthusiastic couple had prepared all the Lachesis since used by the profession and had begun a reliable pathogenesis of one of our greatest remedies.

The idea of thus using virulent animal products as medicines, especially when diluted in alcohol, which was thought to antidote the poison in the blood of the recent victim, had to fight its way. From the time of these initial experiments (1828), for twenty-five years, all manner of theoretical objections were made to this and like medicines.

But when in 1878 (two years before his death) Dr. Hering wished to celebrate the fiftieth anniversary of the introduction of Lachesis by receiving reports of clinical cures by this drug, he was overwhelmed by a mass of corroborations from all over the world, and had he lived would have made a complete monograph of the drug. (See *Guiding Symptoms*, Vol. VI—Lachesis.)

The virus of Lachesis introduced into a wound or injected into a vein produces the most dreadful symptoms, and generally death. The victim of a bite from this serpent falls unconscious to the ground, as if struck by lightning, with vomiting and involuntary stool and urine; the place of the bite becomes gangrenous; hands and fingers swollen and insensible, the in-inflammation extending to the arm and the swelling to the shoulder, with here and there gangrenous blisters; death ensues in a few hours, with loss of consciousness, somewhat irregular motions of limbs, cold, clammy sweat; slow, small, almost imperceptible pulse; expression of pain in the face, which is swollen and puffy. If any reaction at all, there soon appears constant fever, dry, burning skin; constant thirst; dry, coated tongue; small, rapid pulse; weak eyes, anxiety and oppression of the chest; and later, constant sleeplessness, extreme exhaustion; no stool nor urine (for seven days in one case); vertigo, as from intoxication, etc. Post-mortems have shown that the oppression of the chest, with its torturing anguish, is very frequently caused by extension of gangrene to the lungs and liver.

Lachesis poisons the nerve centres, that the whole cerebro-spinal system is affected with the rapidity of lightning is shown above in the sudden dropping of the patient into an unconscious state or sometimes into convulsions; and that the pneumogastric is its special point of attack, is evidenced by the rapidity of the ensuing irritation of the throat, larynx, bronchi, and heart. The provers' symptoms complete our knowledge of the specific action of Lachesis upon the pneumogastric. The throat and laryngeal symptoms of Lachesis are very numerous and distinctive, mainly subjective (except hawking of mucus), and include well-nigh every form of distress from which the parts suffer; dryness, pain, burning, rawness, swelling, dysphagia, worse upon "empty" swallowing than during ingestion, and more from liquids than solids; painful and sensitive larynx; hoarseness with continued tickling cough, and a constant desire to draw a deep breath; all symptoms being aggravated by external pressure, or the mere contact and weight of clothing.

This poisoning soon spreads to the sympathetic, thus involving the entire machinery of life, and causing decomposition of the blood with gangrene. This later, but equally rapid effect, decomposition of the blood, is evidenced not only locally near the bite, but by the hemorrhages from the mucous surfaces eccymoses at various points on the body. The blood loses its fibrin, becomes fluid and oozes devitalized from the tissues. The local results of the bite afford further objective evidences of blood changes. The inflammation set up is always of an asthenic or malignant character, either a cellulitis or an erysipelas, the fluids from which set up abscesses of the adjacent lymphatic glands, inflammation of the areolar tissue along the extremity affected, or of its veins, resulting in pyæmia; locally gangrene quickly ensues, the whole general accompaniments being those of a low typhoid state.

The detailed symptomatology of Lachesis is too long for this paper. The above outline is meant to clear the way for a better understanding of the drug.—*Minneapolis Homœopathic Magazine.*

